# Unmet Needs of Endometriosis Patients with Respect to Health Care Services: A Qualitative Study Using a Patient Training Workshop

**DOI:** 10.3390/jcm14103504

**Published:** 2025-05-16

**Authors:** María Angeles Martínez-Zamora, Georgina Feixas, Eva Palou, Eva Flo, Aida Mallorquí, Meritxell Gracia, Anne-Sophie Gresle, Joan Escarrabill, Francisco Carmona

**Affiliations:** 1 Gynecology Department, Clínic Institute of Gyneacology, Obstetrics and Neonatology (ICGON), Hospital Clinic of Barcelona, Universitat de Barcelona, Institut d’Investigacions Biomèdiques August Pi I Sunyer (IDIBAPS), 08036 Barcelona, Spain; megracia@clinic.cat (M.G.); fcarmona@clinic.cat (F.C.); 2 Gynecology Department, Clínic Institute of Gyneacology, Obstetrics and Neonatology (ICGON), Hospital Clínic of Barcelona, Universitat de Barcelona, 08036 Barcelona, Spain; gfeixas@clinic.cat; 3 Patient Experience Observatory, Hospital Clínic of Barcelona, 08036 Barcelona, Spain; palou@clinic.cat (E.P.); gresle@clinic.cat (A.-S.G.); escarrabill@clinic.cat (J.E.); 4 Endometriosis Association Catalonia, 08018 Barcelona, Spain; evaflo@icm.csic.es; 5 Clinical Health Psychology Section, Psychiatry and Clinical Psychology Department, Institute of Neurosciences, Hospital Clínic of Barcelona, 08036 Barcelona, Spain; mallorqui@clinic.cat

**Keywords:** patients’ experiences, training program, training workshop, healthcare, unmet needs, endometriosis

## Abstract

**Background/Objectives:** Nowadays, endometriosis is considered a chronic inflammatory disease and has a very high impact on women who suffer from it due to the symptoms of pain and infertility, as well as the delay in diagnosis. The objective of our study was to identify the unmet needs of endometriosis patients and to explore opportunities for improvement with respect to health care services for endometriosis patients using a patient training workshop. **Methods:** A qualitative study with a participatory action research method was performed. A two-day training workshop for patients was designed to allow them to develop the confidence and skills necessary to participate in the identification of unmet needs in their healthcare. Eighteen patients were selected by purposive sampling. After each training session, a debate on a topic of interest was also organized among all the participants. Data collection involved the nominal group technique with triangulation of data for their analysis. **Results:** The suggestions brought up during the sessions were divided into four areas of work: improvement of patient care, information and communication, training of professionals/patients, and encouragement of patients to participate. This study is a successful novel example of co-production with endometriosis patients and health care professionals and provides valuable information for future improvements in the care of these patients. **Conclusions**: Co-production between endometriosis patients and healthcare professionals allowed us to identify unmet needs in their healthcare.

## 1. Introduction

Nowadays, endometriosis is considered a chronic inflammatory disease defined by the presence of functioning endometrial-like tissue outside the uterus. Endometriosis affects up to 10% of women in the fertile period and has a very high impact on women with this disease due to the symptoms of pain and infertility, as well as the delay in diagnosis, which can be as long as ten years [1,2]. It has been suggested that the approach to endometriosis should be changed from one that focuses on surgical or pharmacological problems to an approach predominantly aimed at the systemic status of an inflammatory process [1,2].

The healthcare of people with chronic diseases, such as endometriosis, cannot be approached in the same way as in acute diseases, in which the care response is reactive, focused on the problem, and fragmented [2,3,4]. There are several specific models aimed at improving care services for people with chronic diseases. Regarding the design of these models, the literature describes that the best strategy for improving services is to co-produce the identification of unmet needs with the people who receive these services [5]. Co-production with patients helps to better identify unmet needs and facilitate their acceptance and scalability; moreover, the value is defined from the perspective of the person receiving the service and not from those who provide it [6,7]. In addition, integrated care for chronic diseases cannot be organized on the basis of “one size fits all” models, and it is reasonable to assume that the design should consider the potential provision of care according to local availability [5,7].

In relation to the involvement of patients in the improvement of care services, a number of barriers and limitations have been identified that may hinder this process. From the patient’s perspective, the limitations include their beliefs, level of education, or competencies in health, availability of time, or lack of knowledge of opportunities to participate. From the point of view of organizations, there is little knowledge about the methods of participatory processes, or the promotion of patient participation is simply symbolic [5,7].

In-depth studies are still required to assess the impact of patient engagement on improving services and outcomes. However, the process of generating this evidence should not serve as an excuse for not answering a key question: How do we want to care for our patients? [6,7].

In this context, the Gynecology Department of a tertiary hospital, a referral center for endometriosis patients, aimed to improve care services for patients with endometriosis through their active participation in the design and evaluation of these health care services. The objective of our pilot study was to establish a feedback mechanism that allows the identification of unmet needs with respect to health care services in endometriosis patients.

## 2. Materials and Methods

A qualitative study was designed from a multidisciplinary perspective. The participatory action research method [8] was used according to the phases presented by Guba and Lincoln under the constructivism paradigm [9].

The study was approved by the local Ethical Committee, according to prevailing regulations (Reg. HCB/2021/1333; 20 May 2022). Oral and written informed consent was obtained from all participants. The study was structured into two distinct parts: a first part that consisted of a two-day training workshop organized to provide patients with the skills to participate, and a second part that consisted of creating debate sessions that allowed patients to describe their perspectives and preferences and prioritize key issues with respect to unmet needs related to their experience with the healthcare system. Finally, the patients were asked to evaluate the sessions using an online survey.

Data collection involved nominal group techniques and triangulation of data for the analysis, and used a questionnaire to increase the quality, scope, and depth of the study [9,10].

Patients were invited to participate through purposive sampling. To capture the clinical and epidemiological variability among endometriosis patients, we invited participants based on factors such as age, pain symptoms, time since diagnosis, and treatment. Specifically, we included patients in three age groups: under 35 years old (n = 6), between 35 and 45 years old (n = 6), and over 45 years old (n = 6). At least half of the participants in each age group were required to have chronic pelvic pain, a diagnosis made within the last 24 months, and to be undergoing hormonal treatment. Exclusion criteria included a history of current or past malignancies, endocrine disorders, cardiovascular diseases, and other systemic illnesses. The practical information of the study was sent by email by the Endometriosis Advanced Practice Nurse to patients with endometriosis treated in our department. All those interested in participating in the study were able to do so by sending their contact details to the organizing team. The first 18 patients who answered the email, fulfilled the inclusion and exclusion criteria, and could attend the scheduled sessions were invited to participate as a focus group in the two programmed sessions of the activity. All patients gave informed consent to participate in the activity.

Taking into account possible difficulties that patients might encounter in participating, we considered that the training program should start with specific training on the functioning of a hospital, organizational barriers and restrictions, and opportunities for participation. Therefore, we designed a training workshop for patients that allowed them to develop the confidence and skills necessary to participate and actively engage in this process [11]. The training activity was proposed from an open and co-creative perspective to patients with endometriosis treated at our hospital. The course was taught in two sessions of two and a half hours at the continuing education center of the hospital. The two sessions were carried out over two consecutive weeks. The workshop was attended by a multidisciplinary training team composed of professionals from different fields: health professionals from the Endometriosis Unit, professionals in the evaluation of patient experience, and members of the management team of the hospital and the Agency for Quality and Health Evaluation (AQuAS; Agència de Qualitat i Avaluació Sanitàries de Catalunya). A person with another chronic disease who described her collaboration with the hospital, as well as a woman with endometriosis with long-term follow-up in our hospital, participated as facilitators. The content of the workshop was designed with the aim of providing theoretical and practical knowledge to the participants on (1) the participation of patients and users in processes to improve health care, (2) the functioning of the hospital and health services in the national health system, and (3) practical examples of projects and initiatives carried out with the participation of patients. The content of the training program is described in Table 1.

In relation to co-production with patients, in each training session, a debate was also organized among all the participants on a topic of interest. In the first session, the debate focused on the possible barriers that may exist and involve patients in the health system. In the second session, the debate focused on the responsiveness of patients with endometriosis within the health system.

Throughout the two sessions, the workshop organizing team recorded the needs expressed by the participants, as well as their ideas and suggestions.

This collection of information and suggestions was done by nominal group techniques, writing the ideas that were emerging on two posters. An online form was also given to participants during the first two sessions so that they could submit their opinions and comments outside of course hours.

An additional work session was organized with the participation of patients, with the aim of collecting the suggestions that patients had made during the two sessions of the course and prioritizing these suggestions according to their degree of feasibility, importance, and urgency.

After presenting the synthesis of the needs and suggestions collected from the patients, five areas of prioritization were identified. Afterwards, the participants voted on the priority issues within each area. In the last part of the session, the aspects highlighted by the patients were discussed, with the aim of agreeing on degrees of importance and feasibility among the group.

Using the dynamics described above, patients were able to define their perspectives and preferences and prioritize key issues regarding unmet needs related to their experience with the health system. Subgroup analysis was performed to examine how age, time since diagnosis, and treatment type influenced needs.

The evaluation of the activity was performed through an online survey sent by email to all participants to assess the level of overall satisfaction with the course, its organization, and its contents. It was sent one week after the last work session. There were also open-ended questions to describe the most valued aspects of the activity, the elements that the participants had missed, and suggestions for improvement for future editions of the activity.

## 3. Results

### 3.1. Participants of the Training Workshop

Eighteen endometriosis patients composed the group. Clinical and demographic characteristics of patients are described in Table 2.

### 3.2. Areas of Work Obtained During the Workshop Sessions

The suggestions developed during the workshop were classified together with the patients into four areas of work: (1) improvement of patient care, (2) information and communication, (3) training of professionals/patients, and (4) encouraging patients to participate. These four general areas were divided into different categories according to each area of work, and specific suggestions were obtained (Table 3).

### 3.3. Elements Prioritized in the Different Work Areas

In the last part of the methodology described, suggestions in all the categories belonging to the four areas were discussed.

Likewise, the prioritization dynamics carried out during the debate and co-production workshop after the course allowed for the identification of the most important elements to be considered in the plan to improve health care for people with endometriosis (Table 4). Subgroup analysis showed that patients younger than 35 years, those diagnosed within the last 24 months, and those undergoing hormonal treatment prioritized ‘Information and Communication’. Participants older than 35 years, those diagnosed more than 24 months ago, and those not undergoing hormonal treatment prioritized ‘Improvement in Patient Care’. Specifically, patients under 35 years old, those receiving hormonal treatment, and those diagnosed in the last 24 months emphasized the importance of the ‘Overview’ category and requested more information regarding sex life, fertility, and disease understanding. Patients older than 35 years and those diagnosed more than 24 months ago were more focused on the ‘Organization’ category, highlighting the need to facilitate access to other specialists and organize multidisciplinary consultations.

### 3.4. Online Survey of the Activity Evaluation

Analysis of the results of the activity evaluation survey showed a high level of satisfaction among the participants (83% described a high or very high level of satisfaction with the course in general). Organizational aspects, such as duration, site, and previous communications, were also assessed positively, and it was stated that the information had been relevant and useful. However, participants also indicated the need to specify the objective of each session more clearly, as 33.3% felt that the description given had not been sufficiently clear and precise.

In the answers to the open question of the questionnaire (“Do you want to give any suggestion(s) to improve the future activities that we will organize?”), the highlighted aspects were the participatory methodology of the course, the possibility of interaction with the speakers and between participants, the fact of being able to share the experiences lived with other affected women, to be part of a participatory project to propose improvements and help women affected in the future, and the desire to participate and involvement of the professionals involved in the course.

Among the aspects to be improved or considered for future editions, the following were mentioned: the extension of the time dedicated to the debate and the voicing of opinions, experiences and suggestions by the participants, the organization of shorter sessions with the possibility of connecting online, and the fact of being able to also count on the participation of those affected during the organization of the workshops.

## 4. Discussion

In the present study, we aimed to address improvements of care services for patients with endometriosis, for the first time, through their active participation in the design and evaluation of these care services. Nowadays, there is no doubt about the impact of endometriosis on the daily lives of patients and that there are multiple aspects of the emotional impact of the disease that need to be studied in depth [1,12,13].

We were aware that the solutions proposed in this study would have to be explored, at least first, at the local level and on the basis of specific needs and the resources available. For this reason, it was considered that the starting point should be specific training within the operation of a hospital, organizational barriers and restrictions, and opportunities for participation [7]. Co-production with endometriosis patients allowed us to identify the specific unmet needs in the healthcare of these patients. The suggestions made during the course were developed around four areas of work described jointly with the participating patients: improvement of patient care, improvement of information and communication, improvement of training of professionals and patients, and encouragement of patient participation. The subgroup analysis based on age, time since diagnosis, and treatment type revealed differences that will allow interventions to be tailored more precisely. ‘Information and Communication’ and ‘Improvement in Patient Care’ were prioritized. Younger patients, those diagnosed more recently, and those receiving hormonal treatment requested more information about the disease and its management. Older patients, those without hormonal treatment, and those diagnosed longer ago prioritized organizational aspects and resources. Therefore, strategies to address unmet needs should consider patients’ clinical and epidemiological characteristics to enhance care. These preliminary findings may allow for future development of care innovations that can be implemented in the short or medium term [7].

Few previous reports have underlined the importance of taking into account the unmet needs of endometriosis patients [3,14] and their barriers to participation and research priorities [15]. These studies pointed out that the evaluation of unmet needs in women with endometriosis would facilitate the design of patient-centered interventions to meet these needs and ultimately improve quality of life, and that people with endometriosis were open to participating in research that they felt aligned with their needs [15,16,17]. In the last few years, several specific models focused on improving care services for people with chronic diseases have been proposed [7,18,19]. Regarding the design of these models, the literature describes that the best strategy to propose for the improvement of services is to co-produce improvements with the people who will receive them [7,19]. Internationally, patient participation in the development of innovations in the field of health is articulated around conceptual frameworks such as “patient advocacy”, defined as the set of efforts to offer support to the patient to ensure their interests and safety [20,21,22,23]. In some countries, such as France, there have been changes in legislation that favor the participation of patients within the framework of what is called “democratic health”. This consists of training programs for patients developed in various universities to give them the skills to act as patient representatives, and the “experiential knowledge” of patients is recognized and legitimized [7]. In Quebec, they have developed the patient partenaire model to promote the participation of patients who are involved in continuous quality improvement activities and favor multiple training activities for patients [19]. Furthermore, the Ottawa model focuses on involving patients and families in research projects to design these projects around patients’ needs and make them reproducible and scalable [20]. Along the same line, the Mayo Clinic has training programs to promote the participation of patients in the development of clinical practice guidelines [22] and the European Patients’ Academy on Therapeutic Innovation (EUPATI), a non-profit foundation, has training programs for the participation of patients in the research and development of medicines [23].

In relation to the participation of patients in the improvement of care services, a series of barriers and limitations have been detected that may hinder this process. The patient’s perspective limitations include their own beliefs, level of education, availability of time, or lack of knowledge about opportunities to participate; from the point of view of organizations, having little knowledge about methods of participatory processes or the fact of promoting the participation of patients in simply a symbolic way must be considered [24]. However, sometimes small organizational changes related to the real needs of patients can achieve significant results [25].

Innovations in service delivery can be generated in various ways and through various mechanisms [26]. Sometimes, the trigger is a new technology or treatment. Another mechanism may be an organizational idea or the need imposed by some studies in the institution that provides the service. However, there is another engine to promote innovations in the provision of a service, which is to identify the unmet needs of the people who will receive this service. In all three cases, the participation of patients is very important, but if this occurs, it rarely happens from the beginning of the project.

From our experience, improvement in service delivery is an iterative process that must be adapted to the local circumstances of the clinical practice in which it is implemented. The fundamental principle of this approach is not to make assumptions [27].

Our study has several strengths. First, we designed the training program before the workup of the focal group; therefore, patients could participate adequately. Second, all patients had been diagnosed with endometriosis and had been treated in a specialized unit of endometriosis; therefore, they had previous experience as patients in this specific area of healthcare. Finally, the study design and the number of participants provide an initial overview of the variety of patients’ needs and their priorities.

Nevertheless, our study has some limitations. On the one hand, the qualitative research of the present study does not provide evidence-based recommendations. On the other hand, the selection of patients from the database of a tertiary referral center for the care of endometriosis patients may be a source of potential bias and may limit the generalization of our findings, which may be more pertinent to practices providing high-volume endometriosis care and in regions with healthcare delivery similar to our geographical area.

This novel work lays the foundation for further research. Future steps may include the development of a platform for monitoring patient interactions, enhancing quality of life, and enabling continuous adaptation through real-time feedback. Such a study should allow for comparisons between cohorts of patients exposed to different interactive approaches or algorithms in order to identify the most effective strategies. In addition, the design of tailored information tools for different patient profiles is essential to address relevant unmet needs. Finally, the evaluation of implemented actions is critical to gather feedback and guide the prospective development of future improvements.

To conclude, the present study describes a specific methodology using a patient training program to allow women with a chronic disease, such as endometriosis, to participate in the exploration of their needs and experiences. This study is a successful novel example of co-production with endometriosis patients and also provides valuable information for the future improvements of health care for these patients. Our study showed that improvements in healthcare services for patients with endometriosis are warranted and underline the unmet needs of endometriosis patients in healthcare services, with the improvement of four areas being notable: patient care, information and communication, training of professionals and patients, and encouragement of patient participation.

## Figures and Tables

**Table 1:** Training Program Content.

First Day of Workshop	Second Day of Workshop
1. The concept of person-centered care.	1. Oral and interpersonal communication skills for participation.
2. The role of the active patient.	2. The operation and governance of health centers.
3. Objectives and methodologies of participation: focus groups and interviews.	3. The financing of health services.
4. Experiences of patient participation in our hospital.	4. Strategies for prioritizing resources (financial, human, etc.).
	5. The concept of shared decisions.

**Table 2:** Demographic and clinical data of the participants.

Demographic and Clinical Data	Endometriosis Patients n = 18
Age	32-52 ^1^
Time since diagnosis	1–8 ^1^
Chronic Pelvic Pain	8 (44.4) ^2^
Previous endometriosis surgeries	10 (55.5) ^2^
Menopausal status	2 (11.1) ^2^
Under hormonal treatment	14 (77.8) ^2^

^1^ Range in years. ^2^ [n (%)].

**Table 3:** Four main work areas, classification of categories, and patient suggestions.

Work Areas	Categories	Patient Suggestions
**1. Improvement of ** **patient care **	First Visit	- Improve information received during the first visit. - Explain the functioning of the Endometriosis Unit. - Explain the disease.
	Organization	- Improve access to diagnosis. - Facilitate access to other specialists (nutritionist, osteopaths). - Arrange the visit with doctors the same day after transvaginal ultrasound. - Organize multidisciplinary consultations.
	Tracking	- Improve follow-up and discharge. - Facilitate referral to complementary services
	Pain	- Mitigate pain during gynecological tests with relaxation or sedative techniques.
	Psychological support	- Offer psychological support, with special attention during hormonal treatments.
	Other aspects	- Asses and inform about alternative treatments. - Improve disease monitoring even after menopause
**2. Improvement in** **information and** **communication**	Overview	- Provide information on: - The disease. - Fertility and sexual life. - Life with endometriosis. - The genetic factor. - Research into endometriosis.
	Testimonies	- Review of informative support communication of the hospital that is better focused on hope and a positive message. - Teach the diversity of situations and patient profiles. - Explain the experiences lived. - Develop a guide to living with endometriosis.
	Unit Information	- Provide information on the functioning, contact, and care team - Provide a welcome document with general information about the disease and information about the Endometriosis Unit.
	Environment	- Provide information to the family/cohabitants and social environment of patients. - Organize discussion groups for family/cohabitants and the social environment of patients.
	Shared Decisions	- Provide information to facilitate shared decision-making.
**3. Improvement in** **training of ** **professionals and** **patients**	Patient Training	- Create a course for all patients that includes: general aspects of the disease, its impact on everyday life, sex life, the function of the Unit, and the usefulness of the investigation developed.
	Training for Health Professionals	- Organize workshops for professionals on the importance of mutual listening and empathy. - Improve training in non-pharmacological treatments. - Organize training in the practice of transvaginal ultrasounds. - Raise awareness among professionals about endometriosis.
**4. Encourage patient** **participation**	Testimonies	- Share testimonials about the experiences lived with the disease on the website of the hospital.
	Participation in care	- Encourage the participation of patients in working groups. - Encourage patient participation in clinical trials.
	Volunteering	- Create support and discussion groups among patients. - Create volunteer opportunities to accompany patients with endometriosis. - Collaborate with the associations of those affected.

**Table 4:** Elements prioritized in each work area according to the participating patients.

Work Area	Elements Prioritized
**1. Improvement in patient care**	- Facilitate access to other specialists such as nutritionists or osteopaths, among others.
	- Improve information in the first visit: general information on the disease, the service, treatments and their effects, recommendations on nutrition, physical exercise, among others.
	- Facilitate shared decision-making between the patient and professional (inform about different treatment options, treatment side effects).
	- Psychological and/or emotional support and accompaniment when necessary.
	- Improve discharge and follow-up: improve the process of care and contact in the follow-up.
	- Improve disease identification and diagnosis in care networks: provision to outpatient clinics and primary care centers.
**2. Information and communication**	- Improve general information about the disease: intelligible information, treatments, or recommendations to improve quality of life.
	- Improve information on available services and resources, operation, organization, and communication channels.
	- Welcome document with basic information about the Unit and the disease.
	- Information about sex life and fertility.
	- Information on genetic factors.
**3. Training of professionals and patients**	- Create an introductory course for patients.
	- Improve the training of service professionals in aspects such as patient-professional communication, empathy, or mutual listening.
**4. Encouragement of patient participation**	- Promote patient participation in clinical trials and research.
	- Promote patient participation in the development of information materials.
	- Implement and give visibility to support groups and patient accompaniment.

## Data Availability

No new data different than all provided is available in this publication.
